# Sequential study on reactive blue 29 dye removal from aqueous solution by peroxy acid and single wall carbon nanotubes: experiment and theory

**DOI:** 10.1186/1735-2746-10-5

**Published:** 2013-01-05

**Authors:** Mahsa Jahangiri-Rad, Kazem Nadafi, Alireza Mesdaghinia, Ramin Nabizadeh, Masood Younesian, Mohammad Rafiee

**Affiliations:** 1 Department of Environmental Health Engineering, School of Public Health, and center for water Quality Research, Institute for Environmental Research, Tehran University of Medical Sciences, Tehran, Iran; 2 Department of Environmental Health Engineering, Alborz University of Medical Science, Alborz, Iran

**Keywords:** Reactive blue29, Peroxy acid, Adsorption isotherm, Single wall carbon nanotubes, Equilibrium time, Kinetic studies

## Abstract

The majority of anthraquinone dye released to the environment come from antrapogenic sources. Several techniques are available for dyes' removal. In this study removal of reactive blue 29 (RB29) by an advanced oxidation process sequenced with single wall carbon nanotubes was investigated. Advanced oxidation process was optimized over a period of 60 minutes by changing the ratio of acetic acid to hydrogen peroxide, the compounds which form peroxy acid. Reduction of 20.2% -56.4% of reactive blue 29 was observed when the ratio of hydrogen peroxide/acetic acid/dye changed from 344/344/1 to 344/344/0.08 at different times (60, 120 and 180 min). The optimum ratio of acetic acid/hydrogen peroxide/dye was found to be 344/344/0.16 over 60 min. The resultant then was introduced for further removal by single wall carbon nanotubes(SWCNTs) as adsorbent. The adsorption of reactive blue 29 onto SWCNTs was also investigated. Langmuir, Freundlich and BET isotherms were determined and the results revealed that the adsorption of RB29 onto SWCNTs was well explained by BET model and changed to Freundlich isotherm when SWCNTs was used after the application of peroxy acid. Kinetic study showed that the equilibrium time for adsorption of RB 29 on to SWCNT is 4 h. Experiments were carried out to investigate adsorption kinetics, adsorbent capacity and the effect of solution pH on the removal of reactive blue29. The pseudo-second order kinetic equation could best describe the sorption kinetics. The most efficient pH for color removal (amongst pH=3, 5 and 8) was pH= 5. Further studies are needed to identify the peroxy acid degradation intermediates and to investigate their effects on SWCNTs.

## Introduction

Dyes are color organic compounds which can colorize other substances. Dyes are extensively used in the textile, leather, paper and other industries. The complex aromatic structure of dyes make them more stable and difficult to be removed from water bodies [1]. Textile manufacturing is one of the largest industrial producer of wastewater characterized by highly fluctuating pH,high chemical oxygen demand (COD), strong color and biotoxicity [2]. It has been estimated that approximately 50% of applied reactive dyes is wasted because of dye hydrolysis in the alkaline dye bath at concentrations in the range of 10-200mg/L [3]. As the regulations world wide have become more stringent, stricter,the effluent of textile and related industries have to be treated before discharging in to the environment. This has resulted in a highly demand for environmentally friendly technologies. Numerous approaches including electrochemical oxidation [4], ozone treatment [5,6], biological treatment [7], membrane filtration [8], and adsorption [9] have been applied to remove organic compounds. Promising results have been achieved using advanced oxidation processes(AOPs) for effluent from dye industries in recent years [10]. These processes are based on the production of highly reactive radicals, especially hydroxyl reactive radicals which promote destruction of the target pollutant until mineralization [11].

**Figure 1:**
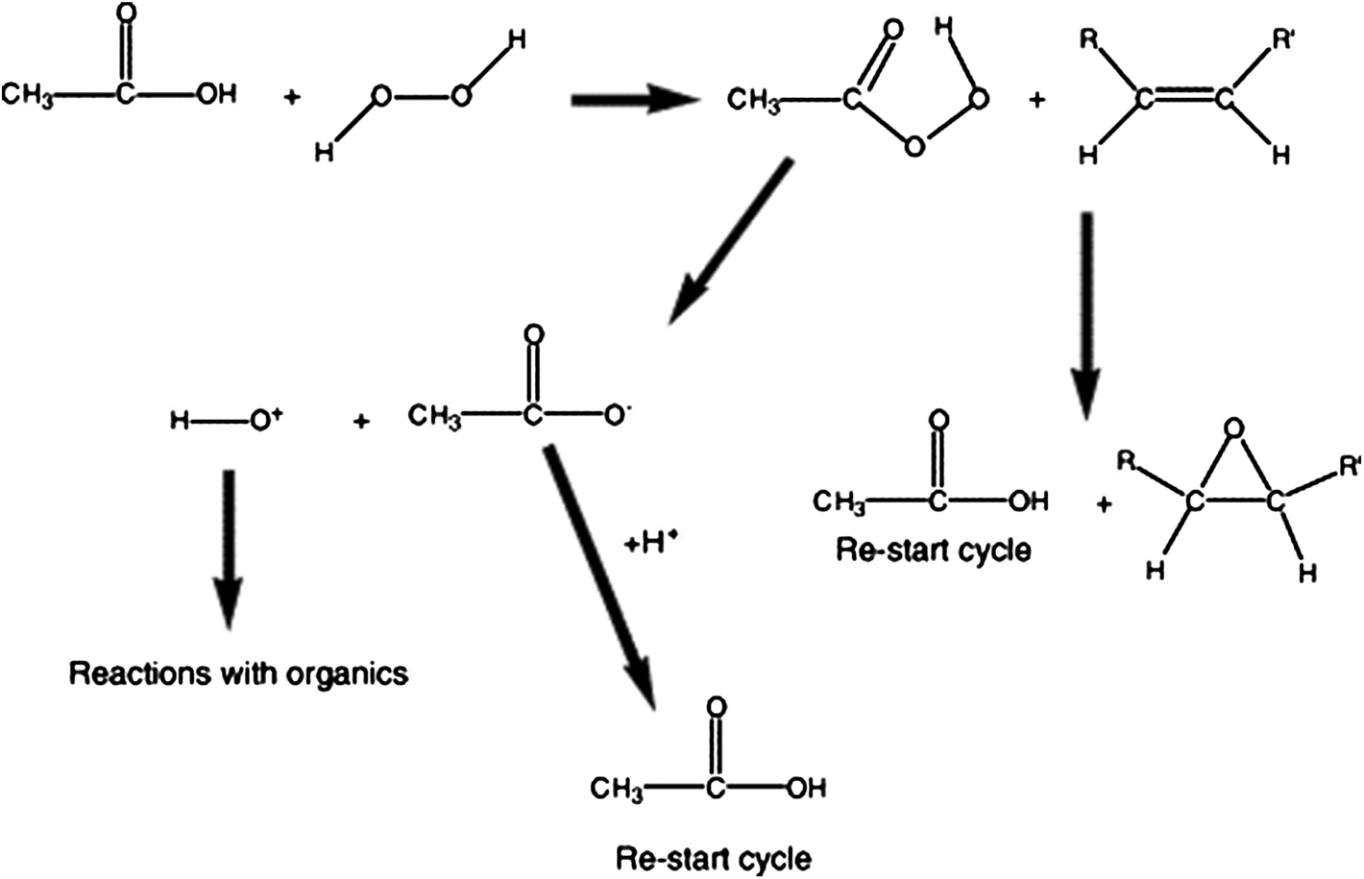
The hypothesized peroxy-acid cycle.

**Figure 2:**
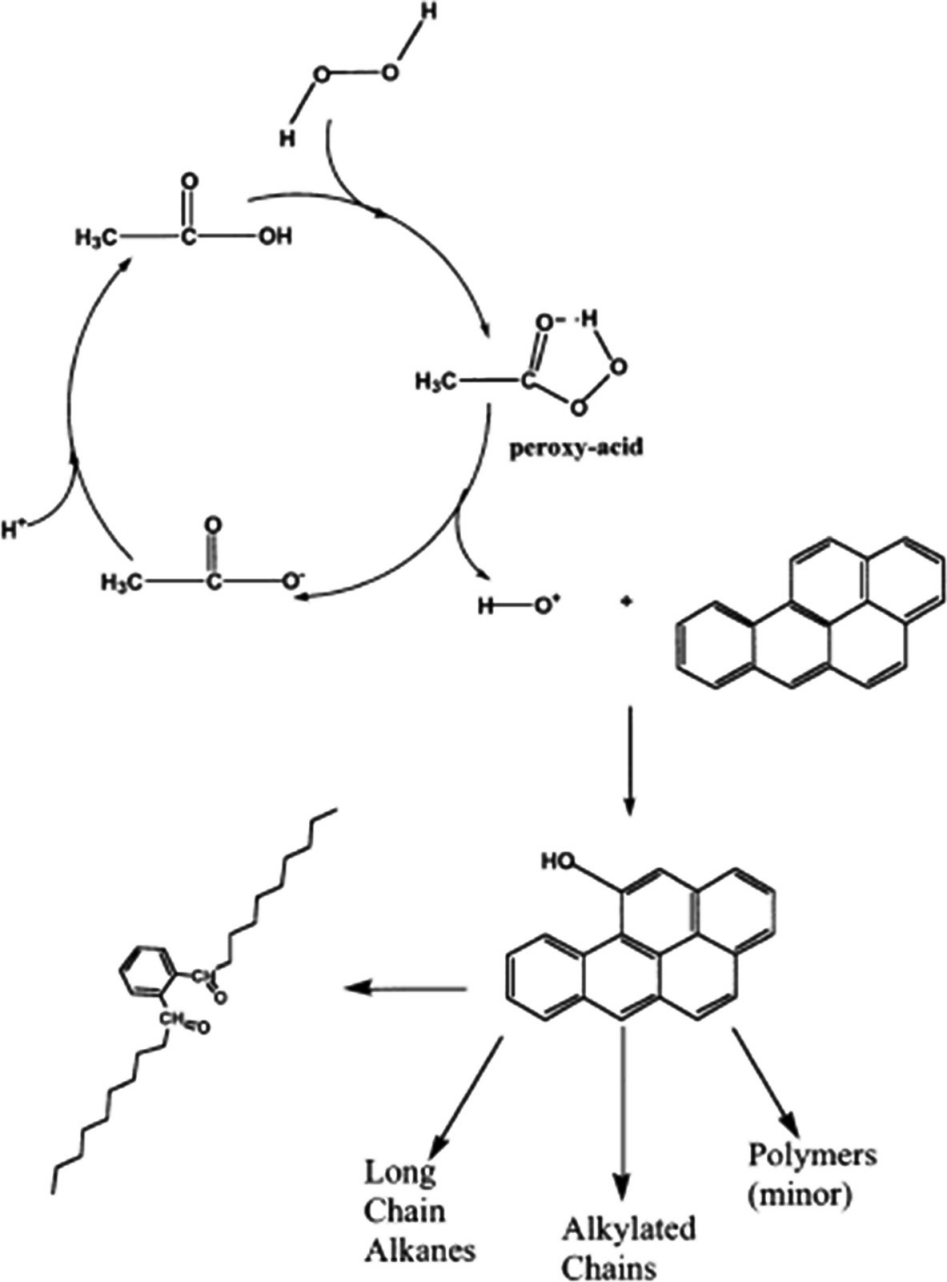
Hipothesized peroxy acid process with banzo[a]pyrene as a model contaminant.

Peroxy acid oxidation occures when acetic acid reacts with hydrogen peroxide which results in the formation of hydroxyl radicals. It has been suggested that peroxy acid oxidation occurs through the formation of hypothesized peroxy acid compounds as illustrated in Figure 1[12] demonstrated the epoxidation of alkanes using peroxy acids. This peroxy acid compound will initiate the release of hydroxyl radical or hydroxyl cation. Like in other AOPs the hydroxyl radical or possibly hydroxyl cation oxidizes the contaminant (e.g.,benzo[a]pyrene in Figure 2). Acetic acid has been determined to be the preferred organic acid to use from previous experiments [13]. In a study by Belgin *et al.*[14] some organic and inorganic intermediates were generated during the degradation of RB4 dye by peroxy acid. The partial charge showed that OH radical attacks to N position on the RB4 to form three intermediates groups alkylated chains, long chain alkanes, and minor polymers.

Moreover, adsorption technology with no chemical degradation is attractive due to it's unique Effectiveness, efficiency and economy [15]. Carbon nanotubes are increasing used in researchs as new adsorbents. They are an alternative for the removal of organic and inorganic contaminants from water because theyhave large specific surface area, small size and hollow, layered structure [16]. According to the grapheme layer, CNTs are classified into sigle wall (SWCNTs) and multi-wall (MWCNTs). Carbon nanotubes are unique and one-dimensional macromolecules which exhibit considerably thermal and chemical stability [17]. More recently, Long and Yang, reported that MWCNTs could be more efficient for the removal of dioxin than activated carbon [18]. Cai *et al.,* prepared a CNT-packed cartridge for the solid-phase extraction of compounds such as bisphenol A and 4-*c*-nonylphenol in environmental water samples [19]. CNTs have been proven to possess great potential as superior adsorbents for removing many kinds of organic and inorganic including 1,2-dichlorobenzene [20], trihalomethanes [21] and cationic dyes [22]. Therefore, CNTs might be ideal sorbents for the removal of dyes from water.

**Figure 3:**
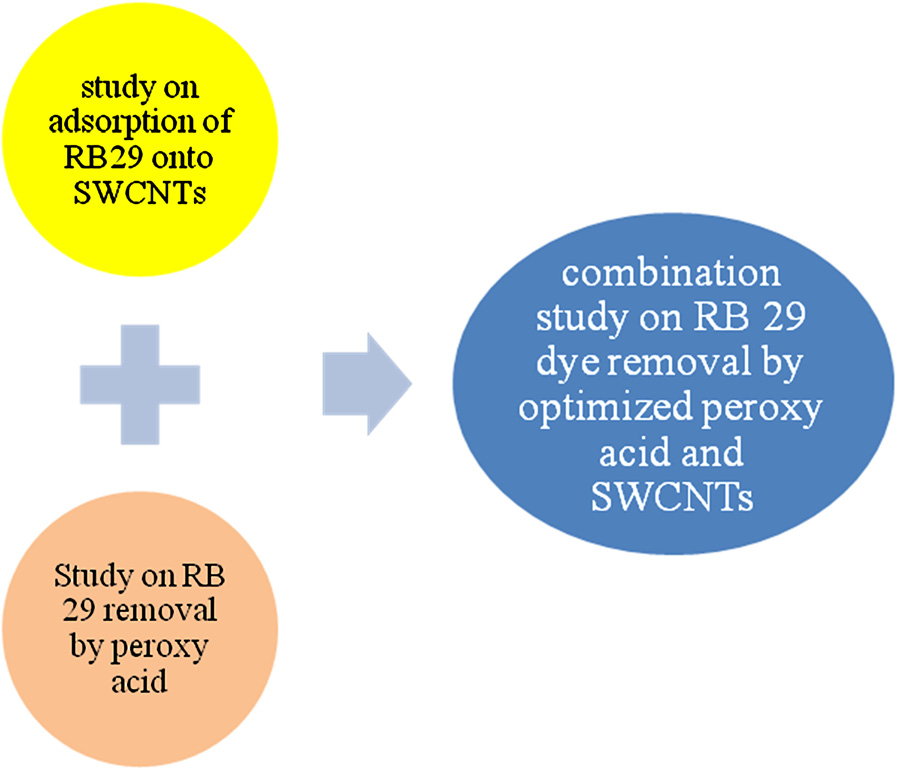
Experimental procedures.

An alternative strategy may be to integrate physical and chemical degradation of organics. This hybrid technology is becoming an accepted sequential practiced,used not only for industrial and hazardous waste but for other organics naturally found in drinking water [23]. In this study the oxidative degradation of a reactive antraquionone dye(RB29)in aqueous solution using peroxy acid followed by single wall carbon nanotubes (SWCNTs) as adsorbent was investigated(schematicly shown in Figure 3).

Composite textile wastewater is characterized mainly by measurements of biochemical oxygen demand (BOD), chemical oxygen demand(COD), suspended solids (SS) and dissolved solids (DS). According to other studies, COD values of composite wastewater are extremely high compared to other parameters. In most cases BOD/COD ratio of the composite textile is around 0.25 that implies the wastewater containing large amount of non biodegradable organic matter.

## Materials and methods

**Figure 4:**
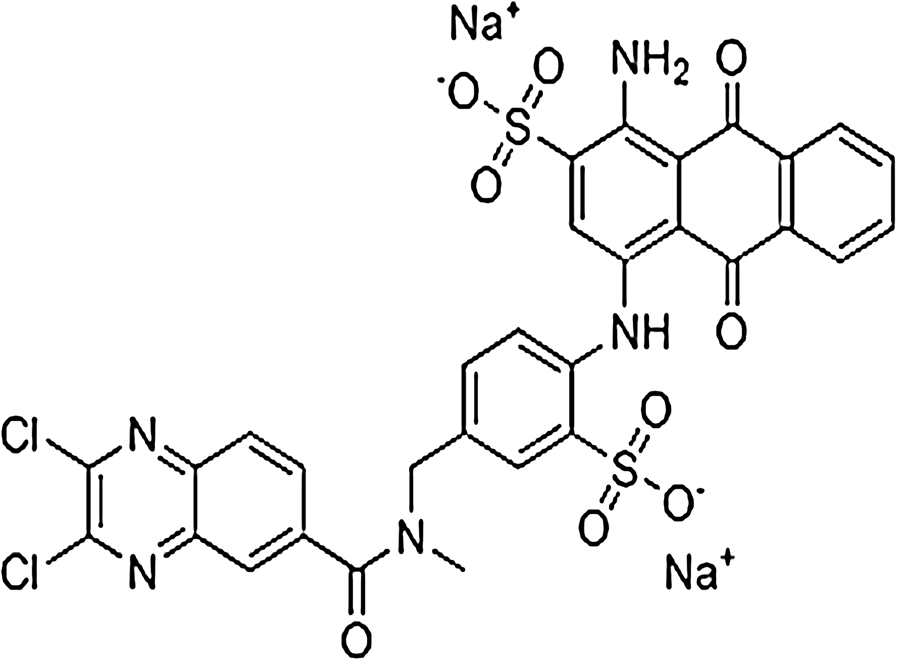
Chemical structure of reactive blue 29 (RB29).

Commercial reactive blue 29 dye was obtained from dystar Hoechst corporation. The formula, molecular weight and maximum wave length of light absorbed by reactive blue 29 were C_31_H_19_O_9_N_5_S_2_Cl_2_Na_2_, 788 g/mol and 589 nm respectively. The structure of reactive blue 29 is shown in Figure 4. All solutions were prepared using deionized water and reagent grade chemicals. Glacial acetic acid, hydrogen peroxide and anhydrous sodium solfite were purchased from Merck company. The single–wall carbon nanotubes were purchased from Iranian Research Institute of Petroleum Industry (R.I.P.I). The solution of RB29 was prepared in 30 mg/L initial concentration for all experiments.

### Characterization of SWCNTs

Single–wall carbon nanotubes were subjected to energy dispersive spectrometer for surface distribution of elemental composition and scanning electron microscopy (SEM). Size and morphology of SWCTs were reported by transmission electron microscopy (TEM). The specific surface area of SWCNTs were measured by BET method. The outer and inner diameter of SWCNTs were 1–2 nm and 0.8-1. 1 nm, respectively. The length of SWCNTs was 10 μm with specific surface area of 700 m^2^/g. The purity of selected single-wall carbon nanotubes was 95%.

### Peroxy acid oxidation

Peroxy acid oxidation experiment were conducted using 250 mL pyramid glass bottles with the addition of 100 mL reactive blue29 (30 mg/L)solution. Predetermined volumes of acetic acid (50%) and hydrogen peroxide (30%) were added to each bottle in order to keep the mole ratio of hydrogen peroxide/acetic acid/dye in 344/344/1 and lower. In other studies degradation of organic matter were investigated by peroxy acid and the range of hydrogen peroxide/peroxy acid/pollutant was kept in the same range [24,25]. The samples then were put on the illuminated refrigerated incubator shaker (Innova 4340) and shaken at 150 rpm, the temperature kept at 318^0c^. The samples were shaken for a chosen reaction time after which the solusion was quenched by adding 1 mL of sodium solfite (1M).

The experiments were carried out for four different mole ratios of hydrogen peroxide/acetic acid/dye (344/344/1, 344/344/0.3,344/344/0.16 and 344/344/0.08) and at various times (60,120 and180 min). The initial and final concentrations and pH of dye was measured by spectrometer and pH meter,respectively. Four control groups experiments were also prepared. Controls did not contain any acetic acid. In order to give some more information concerning the effectiveness of the AOP using peroxy acid, Table 1 shows the reaction rates for reactive blue 29 dye in different mole ratios. Reported correlation coefficients (R^2^) were based on the linear regression of ln dye (C/C_0_) versus time.

**Table 1:** Estimated reaction rates for reactive blue 29

**Mole ratio**	**K(1/h)**	**R**^**2**^
344/344/1	0.23	0.923
344/344/0.33	0.32	0.929
344/344/0.16	0.43	0.959
344/344/0/08	0/46	0.951

### Batch adsorption experiments

Batch adsorption experiment were conducted after the optimum conditions for advanced oxidation process were found. The pH of the solutions were adjusted to 5 by adding NaoH 0.1N. Different suspensions of SWCNTs (4, 6, 7 and 8 mg) were added to each bottle. The solutions were then put again on shaker and equilibrated at 318^0c^ for 24 h.

At the end of the equilibrium period, the suspensions were centrifuged at 4000 rpm for 10 min for analysis of the dye concentration. The adsorption of reactive blue 29 dye was detected using a spectrometer at 589 nm. Each experiment was duplicated and the results are average values. The amount of adsorbed RB 29 onto SWCNTs was calculated as follows (Eq.1):

(1) $$q=\frac{C_{i}-C_{e}}{m}$$

Where: C_i_ and C_e_ are the initial and equilibrium concentrations, respectively in mg/L, and m is the amount of SWCNTs in mg/L. In order to conduct adsorption experiment of RB29 solely, reactive blue (30 mg/L) was equilibrated by different suspensions of SWCNTs (0.13, 0.1, 0.08, 0.06, 0.04 and 0.02 g/L) at pH=5 and temperature of 318°C for 24 h. At the end of the equilibrium period, the suspensions were centrifuged at 4000 rpm for 10 min and the supernatant was sent for analysis of the dye concentration. The adsorption of RB29.

The advanced oxidation process was conducted for initial dye concentration of 30 mg/L after optimum mole ratio of hydrogen peroxide/acetic acid/reactive blue 29 was found. The resultant was then shaken with SWCNT(0.08 g/L) for 2 h. Total color removal efficiency was finally calculated. The Langmuir, Freundlich and BET isotherms were determined to investigate the adsorption behavior of dye remained in the solution onto SWCNTs after AOP process.

## Results

### Advanced oxidation process by peroxy acid

**Figure 5:**
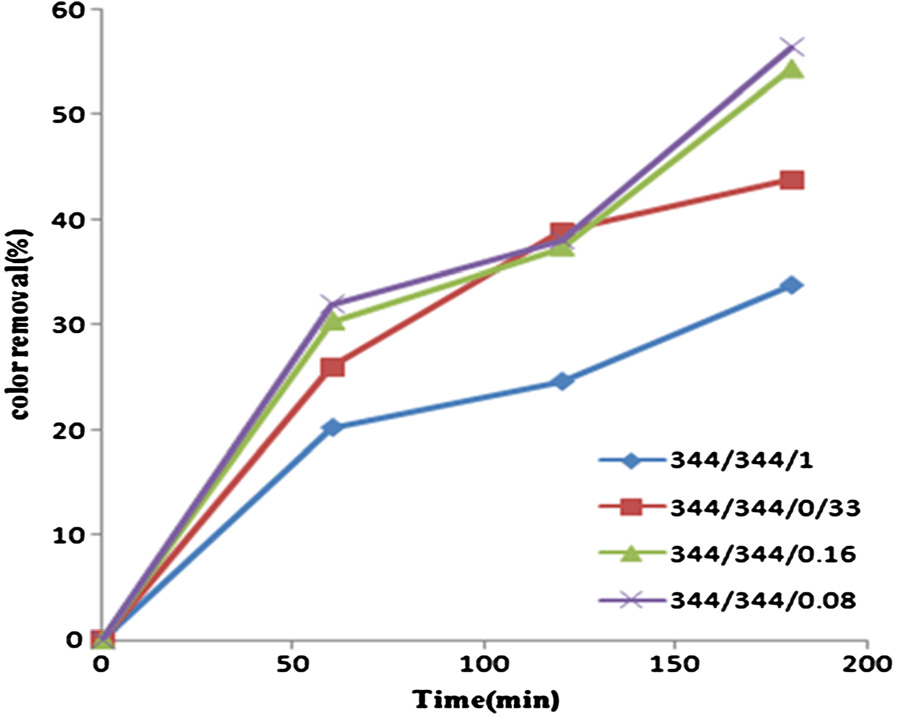
Color removal due to different mole ratios of hydrogen peroxide/acetic acid/dye at various times.

The effects of different mole ratios of hydrogen peroxide/acetic acid/dye at various times (60,120 and 180 min) in color removal are shown in Figure 5, in which by increasing both the mole and time, the color removal efficiency increased. Although the color removal efficiency increased from 20.2%to30% after the mole ratio went up from 344/344/1 to 344/344/0.16, no significant difference was observed by increasing the mole ratio to 344/344/0.08 in 60 min. So the optimum mole ratio was selected as 344/344/0.16 in 60 min. With hydrogen peroxide alone (controls) no significant disappearance of reactive blue 29 was observed (data not shown). An assumption could be made that without the hydrogen peroxide catalyst the AOP process is no more efficient. The final pH of solutions after advanced oxidation process were 2.5-3 depending on the hydrogen peroxide/acetic acid/dye mole ratio. pH of solutions at the end of the reaction was low because of residual acetic acid left in solutions. The reaction rates of RB 29 dye removal by peroxy acid seemed to increase with increasing the mole ratio (0.23/h with the mole ratio of 344/344/1 to 0.43/h with mole ratio of 344/344/0.16) as seen in Table 1.

### Adsorption isotherm

The 1q,LnqandCeCs−Ceq are correlated with the isotherms of models of Langmuir, Freundlieh and BET, respectively:

(2) 1q=1b+1abCe

(3) $$\mathrm{Log}\;q=\mathrm{Logk}+\frac{1}{n}\log C_{e}$$

(4) $$\frac{C_{e}}{\left(C_{s}-C_{e}\right)q}=\frac{1}{A\left(x_{m}\right)}+\frac{A-1}{Ax_{m}}$$

Where C_e_ is the equilibrium concentration (mg/L), b is the maximum adsorption capacity (mg/mg), is Langmuir constant, k and n are Freundlich constants. Xm and A are the amount of solute adsorbed in forming a complete monolayer (mg/mg),and a constant to describe the energy of interaction between the solute and the adsorbent surface in BET model, respectively. Tables 2 and 3 summarizes the coefficient of Langmuir, Freundlich and BET isotherms at pH=5 and temperature of 318K for both experiments.

**Table 2:** Constants of Freundlich,Langmuir and BET isotherm for RB29

**Freundlich isotherm**		
K	n	R^2^
0.225	4.701	0.808
Langmuir isotherm		
a	b	R^2^
1.059	0.4	0.827
BET isotherm		
A	Xm	R^2^
24.52	0.166	0.987

**Table 3:** Constants of Freundlich, Langmuir and BET isotherms for RB29 after peroxy acid process

**Freundlich isotherm**		
K	n	R^2^
0.456	3.036	0.958
Langmuir isotherm		
a	b	R^2^
5.936	0.5	0.908
BET isotherm		
A	Xm	R^2^
260.88	0.451	0.956

### Effect of contact times

**Figure 6:**
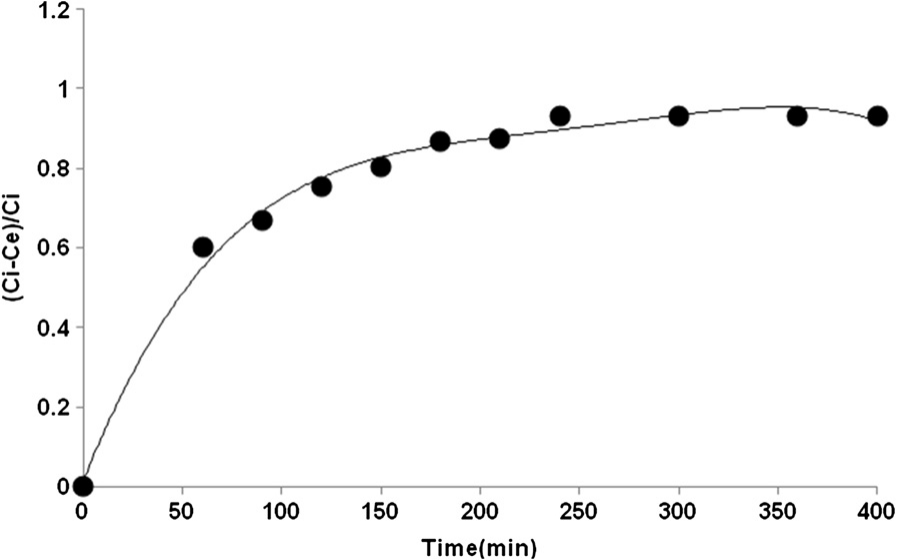
Equilibrium time for reactive blue 29 adsorption onto SWCNT.

250 mL closed pyramid bottles containing 100 mL dye solution and 0.01 g SWCNTs was placed in shaker and shaken at 150 rpm. Different samples were taken every 30 minutes from the bottles in order to find the equilibrium time. Figure 6 shows the effect of contact time on dye removal. For the experiment, the color removal $\left(\frac{C_{i}-C_{e}}{C_{i}}\right)$ increased steeply with time and slowly reached equilibrium, and the reactive blue 29 dye adsorption reached equilibrium in 4 h.

### Kinetics analysis

In order to investigate the adsorption process of reactive blue dyes on SWCNTs,kinetic analysis were conducted using pseudo-first, second and intraparticle diffusion model. The Lagergren rate equation is one of the most widely used adsorption rate equations for the adsorption of solutes from a liquid solution. The pseudo-first-order kinetic model of Lagergren may be represented by

(5) $$\mathrm{Ln}\left(q_{e}-q\right)=1n\left(q_{e}\right){-k}_{1}t$$

where qe and qt are the amounts of dye adsorbed (mg/g) at equilibrium and at time t (min), respectively, and k_1_ is the rate constant of pseudo-first-order adsorption (1/min). The validity of the model can be checked by linearized plot of ln(qe ^_^ qt) versus t. The rate constant of pseudo-first-order adsorption is determined from the slope of the plot. The pseudo-second-order equation based on adsorption equilibrium capacity can be expressed as [26]:

(6) $${t/q=1/k}_{2}{\text{qe}}^{2}{+t/q}_{e}$$

Since neither the pseudo-first-order nor the second-order model can identify the diffusion mechanism, the kinetic results were analyzed by the intraparticle diffusion model to elucidate the diffusion mechanism, which model is expressed as [27]

(7) $${\mathrm{qt}=k}_{i}t^{1/2}+C$$

where C is the intercept and ki is the intraparticle diffusion rate constant (mg/g min^1/2^), which can be evaluated from the slope of the linear plot of qt versus t^1/2^.

The pseudo-first and second order rates and intraparticle diffusion rate equations,values of constants and correlation coefficient for reactive blue 29 removal by SWCNT are shown in Table 4.

**Table 4:** Pseudo-first second order and intraparticle diffusion model parameters

**Pseudo-first order model:ln(q**_**e**_**-q**_**t**_**)=−0.01 t + 5.22**		
q_e_(mg/g)	K_1_(l/min)	R^2^
184.93	0,01	0.95
Pseudo-second order model:t/qt=0.003t+0.16		
q_e_(mg/g)	K_2_(g/mg min)	R^2^
333.3	0.00005	0.983
Intraparticle diffusion model:q_t_=11.6 t ^½^+88.21		
k _i_(mg/g. min ^1/2^)	R^2^	
11.6	0.968	

### Effect of pH

**Figure 7:**
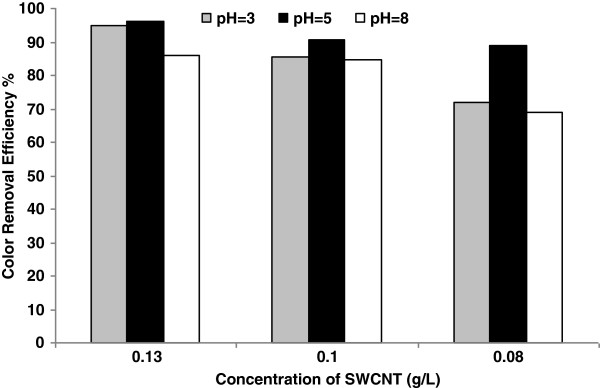
Color removal at different pHs and various suspensions of SWCNT.

Figure 7 shows the effect of pH on color removal in different pHs (3, 5 and 8) and various suspensions of SWCNTs (0/13, 0/1 and 0.08 g/L). It is clear that the pH plays a key role in affecting the adsorption rate of RB 29. Results showed that pH= 5 was the most favorable pH for the adsorption of RB29 followed by pH= 3 and 8 respectively.

## Discussion

### Adsorption experiment

Adsorption isotherm is a dynamic concept when the rate at which molecules adsorb on to the surface is equal to the rate at which they desorb. The equilibrium adsorption isotherm‘s shape is of great importance to provide information about the adsorbents’ surface structure. The data obtained indicated that adsorption of RB29 onto SWCNTs first well explained by BET isotherm with R^2^=0.987 which shows that dye molecules form multilayer on SWCNTs t. Then Freundich isotherm fitted the experimental data better than BET and Langmuir with R^2^=0.958 when SWCNTs were used in sequence with peroxy acid. The Freundlich isotherm model assumes that different sites with several adsorption energies are involved. It is generally known that values of n in the range of 2–10 shows good, 1–2 approximately difficult, and < 1 shows poor adsorption property. SWCNT stated good behavior (n>2) in this experiment. The slope 1/n ranging betwwen 0 and 1 is a measure of adsorption intensity or surface heterogeneity. As this value reaches zero, the surface becomes more heterogeneous. In this study 1/n was < 0.3 indicating the heterogeneity of the surface. R^2^ values of the pseudo-first and second-order models exceeded 0.95 (Table 1), but due to the higher R^2^ values obtained from pseudo-second order, this model represented better adsorption kinetics [28]. Typically, various mechanisms control the adsorption kinetics, the most limiting are the diffusion which include external diffusion, boundary layer diffusion and intraparticle diffusion [29]. When the line of intraparticle diffusion model passes through the origin(C=0), the intra particle diffusion will be the sole rate control step [30]. The regression in our study was linear, but it did not pass through the origin (Table 4), suggesting that adsorption of dye on to SWCNTs involved intraparticle diffusion, but that was not the only rate-controlling step.

### Effect of initial pH

Effect of initial pH is shown in Figure 7. It was observed that dyes adsorbed increased when pH increased from 3 to 5, perhaps suggesting that one of the contributions of SWCNTs adsorption toward cationic dyes resulted from attraction between positively charged reactive blue dye and negatively charged adsorbent surface. As pH increased from 5 to 7, dyes adsorbed decreased. This phenomenon might be resulted from competition between RB 29 dye an OH^-^ on the same SWCNTs sites.

### Combination study of peroxy acid and SWCNTs

The optimum ratio of hydrogen peroxide/acetic acid/dye was found to be 344/344/0.16 in 60 min owing to the higher R^2^ (0.959) calculated for reaction rate (Table 1). This conclusion is based on the future design of the remediation strategy where cost plays a role. In another study conducted by N’guessan, the optimum v/v ratio of peroxide/acetic acid/organic pollutant was determined as 3/5/7 ml [25]. The disappearance of RB29 by peroxy acid seemed to be dependent on the acetic acid as hydroxyl radical formation catalyst and hydrogen peroxide as hydroxyl radicals source. Maximum color removal by combination of peroxy acid and SWCNTs (0.08 g/L) was 66.3%. By comparing the results obtained from adsorption isotherms it is clear that degradation intermediates have direct effects on adsorption of RB29 by SWCNTs because the adsorption isotherm changed (BET in the first stage to Freundlich from in the second). Two assumptions can be made: (a) intermediates compete with RB29 for the adsorption onto SWCNTs or(b) intermediates might inhibit or induce the adsorption of RB29 onto SWCNTs. Mechanism of SWCNTs towards RB29 and AOPs degradation intermediates maybe derivedfrom two reasons. One reason might be based on Van der Waals interactions occurring between carbon atoms and aromatic backbones of the dye and intermediates. The other might be due to the electrostatic attraction between the dye and intermediates onto SWCNTs surface [22]. In another study performed on adsorption of Reactive Blue 4 dye from water solutions by SWCNTs, the same result was obtained, and the authors concluded that Reactive Blue 4 textile dye could be adsorbed on SWCNT through an electrostatic interaction [31]. Both AOP process by peroxy acid and adsorption onto SWCNTs have high affinity for reactive blue29 dye removal. The Freundlich isotherm with R^2^=0.958 well fitted to the data obtained from combination experiment. Maximum color removal was 66.3% in combination process by peroxy acid and SWCNTs (0.08 g/L) as adsorbent. Further research works on testing the effects of intermediates on adsorption of reactive blue29 onto SWCNTs are needed in order to optimize the application of SWCNTs in water treatment.

## Conclusion

Advanced oxidation process and single-wall carbon nanotubes for effective reactive blue dye 29 have been used. Both AOP proces by peroxy acid and adsorption onto SWCNTs have high affinity for RB29 dye removal. The optimum mole ratio of hydrogen peroxide/acetic acid/dye was found to be 344/344/0.16 in 60 min. The adsorption capacity of the adsorbent towards RB29 was illustrated by experimental adsorption isotherms at room temperature. BET isotherm well fitted the data when SWCNT was applied. Experiments were carried out to investigate adsorption kinetics,adsorption capacity of the adsorbent and the effect of solution pH on the removal of reactive blue29. The pseudo-second order kinetic equation could best describe the sorption kinetics. The most efficient pH for color removal was pH= 5.

## Competing interests

The authors declare that they have no competing interests.

## Authors’ contributions

KN, RN and MY participated in the design of the study and performed the statistical analysis. MJR carried out the experimental studies. MR and AM helped to draft the manuscript. All authors read and approved the final manuscript.
